# Supplementary material for: Chromatin remodeling in bovine embryos indicates species-specific regulation of genome activation
Source: Nat Commun. 2020 Sep 17;11:4654. doi: 10.1038/s41467-020-18508-3 (PMC7498599; doi:10.1038/s41467-020-18508-3)
Supplement: Supplementary file 1 — Supplementary Information [file 41467_2020_18508_MOESM1_ESM.pdf]

Chromatin remodeling in bovine embryos indicates species-specific regulation of genome activation

Halstead et al.

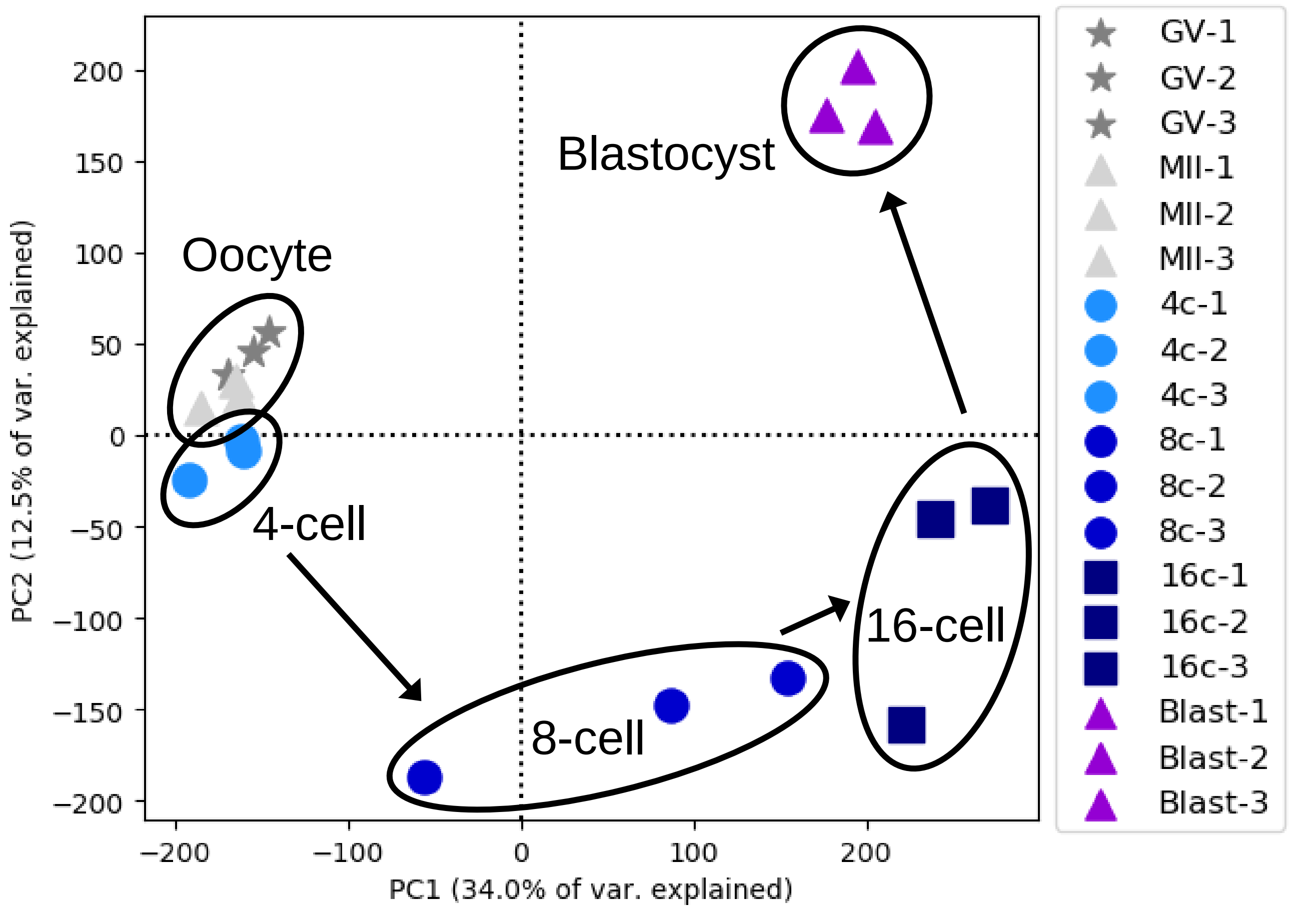

Supplementary Figure 1. Principal components analysis (PCA) of RNA-seq read depth normalized by reads per kilobase million (RPKM) in 50 bp windows covering the whole genome. Sequencing data from Graf et al (2014) [1]. Source data are provided as a Source Data file.

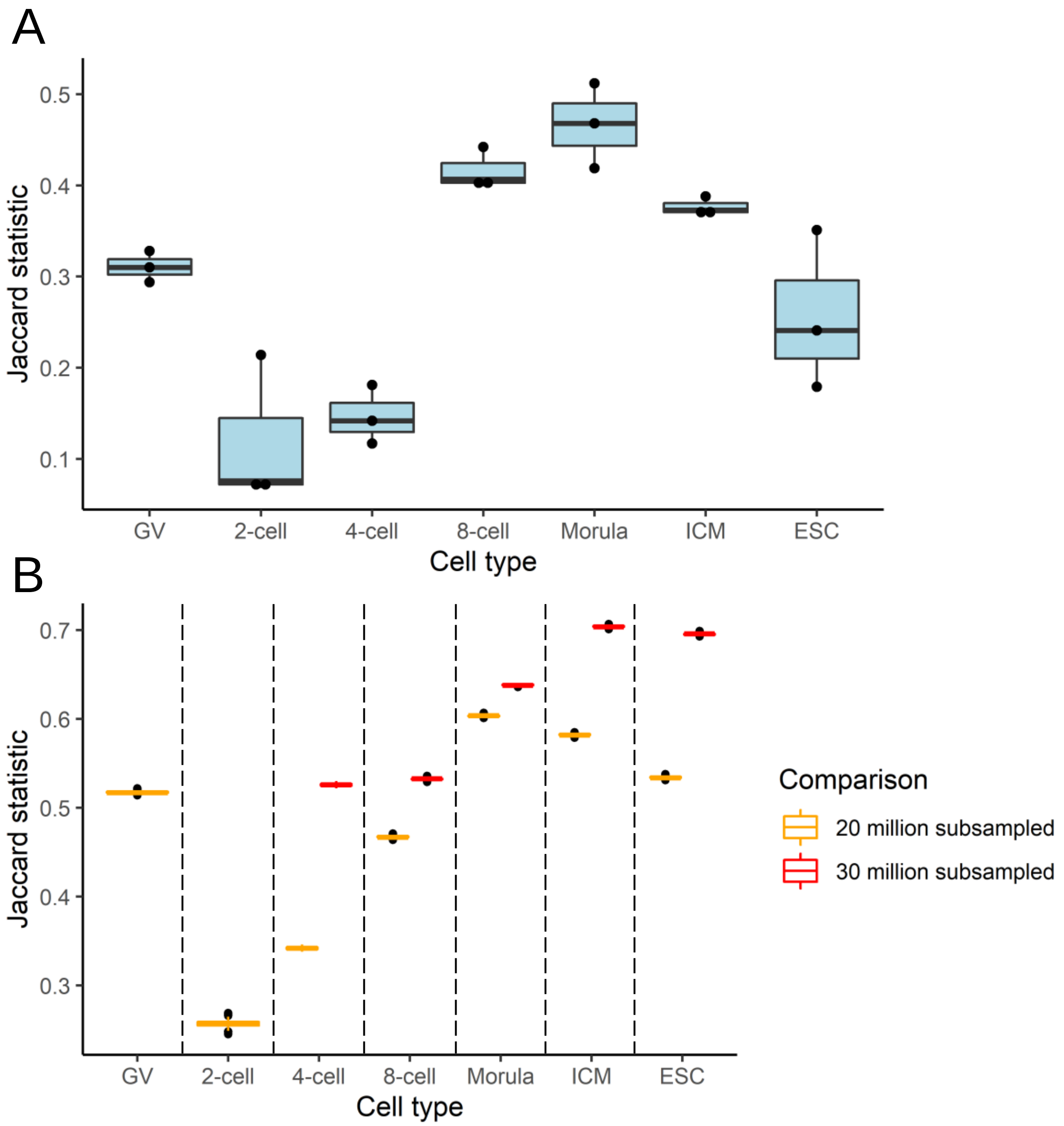

Supplementary Figure 2. Comparison of ATAC-seq peak sets. The Jaccard statistic measures overlap of peak sets, with 0 indicating no overlap, and 1 indicating complete overlap. A) Comparison of peak sets from biological replicates (n=3 biologically independent samples per developmental stage). B) Comparison of peak sets derived from random subsampling from pooled reads for each developmental stage. For each target depth (either 20 or 30 million reads), random subsampling was conducted 100 times, resulting in 100 peak sets that were compared on a pair-wise basis. Boxplots indicate the median and interquartile range (IQR), and whiskers span 1.5 times the IQR. Outliers (data points beyond whiskers) are shown as black points. Source data are provided as a Source Data file.

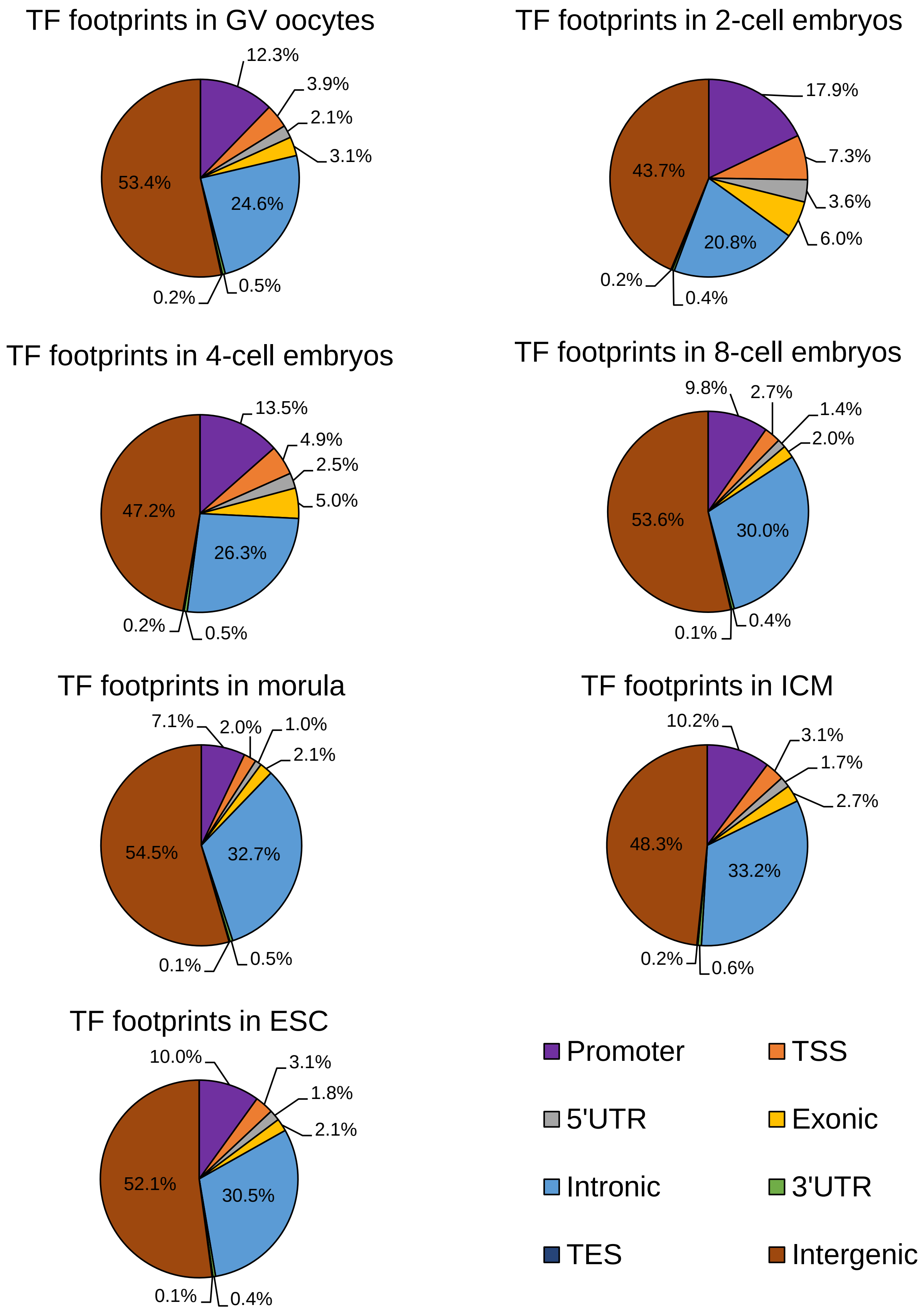

Supplementary Figure 3. Genomic distribution of TF footprints during each stage of development and in ESC. UTR: untranslated region. TES: transcription end site. Source data are provided as a Source Data file.

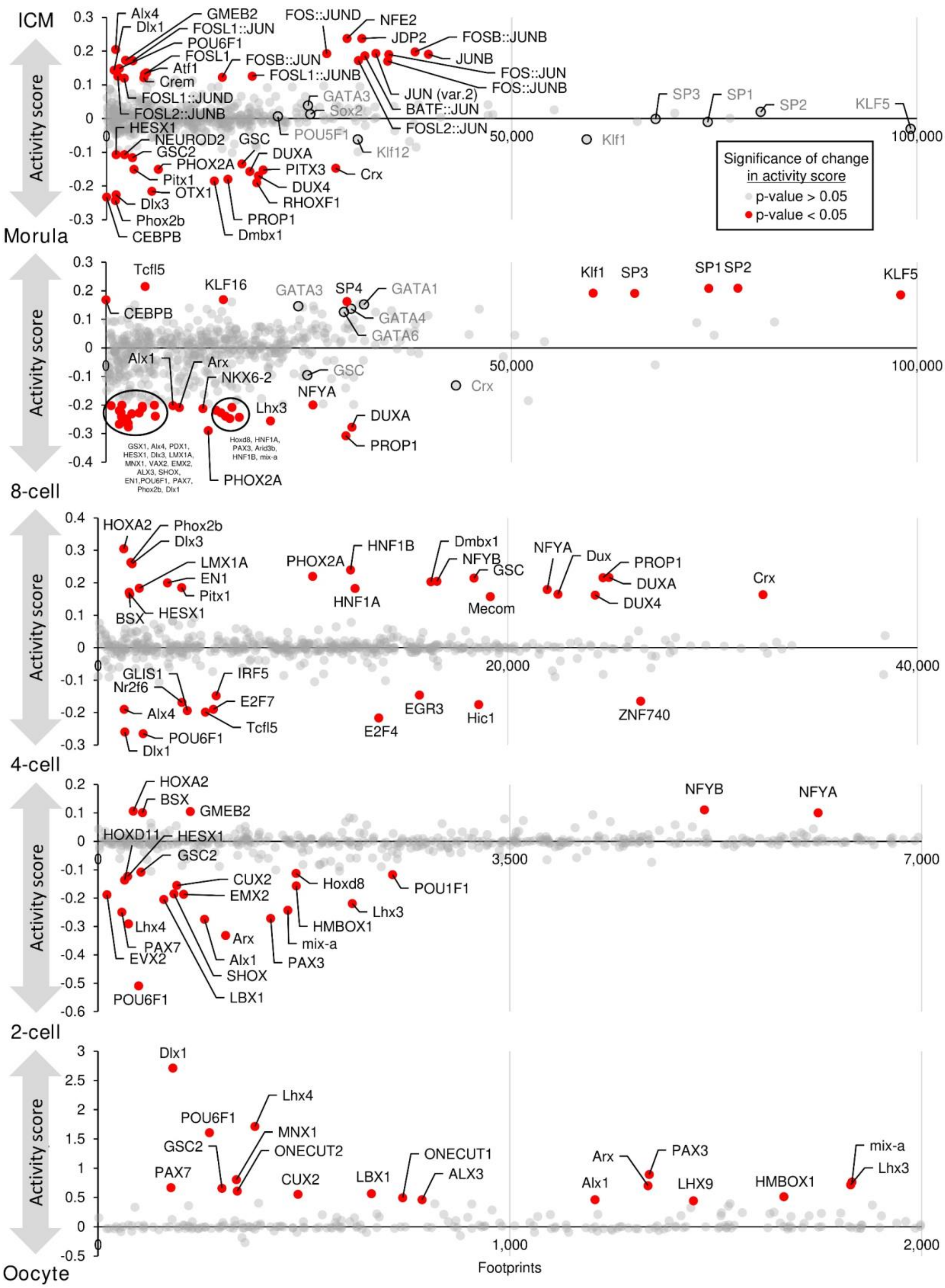

Supplementary Figure 4. Dynamics of transcription factor footprinting during preimplantation development. TF footprint abundance and intensity compared between consecutive stages of development. TFs colored in red demonstrated a significant change in activity between the two stages compared (p-value <0.05; two-tailed t-test). Source data are provided as a Source Data file.

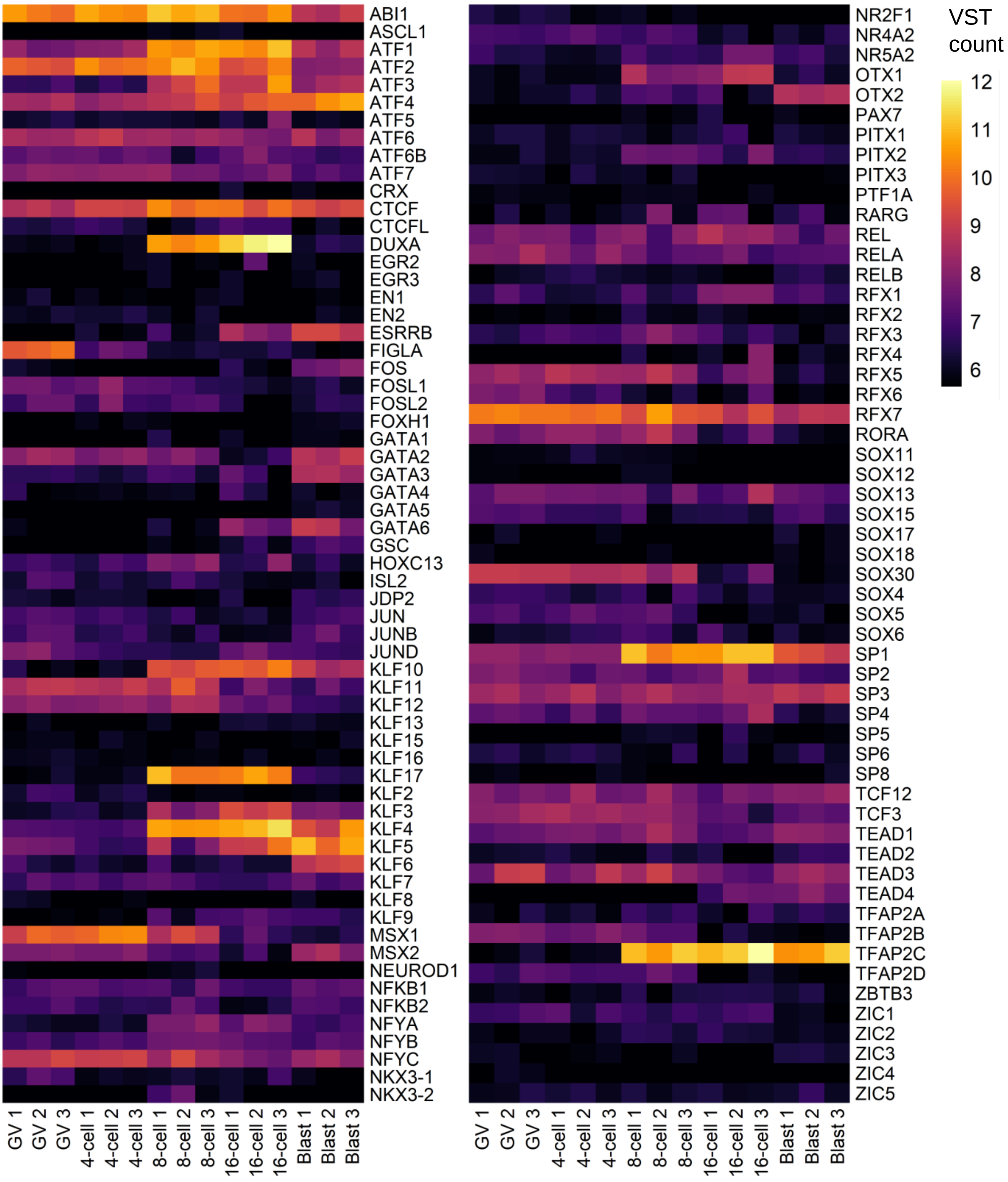

Supplementary Figure 5. Variance stabilized transformed (VST) expression counts for transcription factors with enriched binding motifs in distal open chromatin at some point during bovine preimplantation development. RNA-seq data from Graf et al (2014) [1]. Source data are provided as a Source Data file.

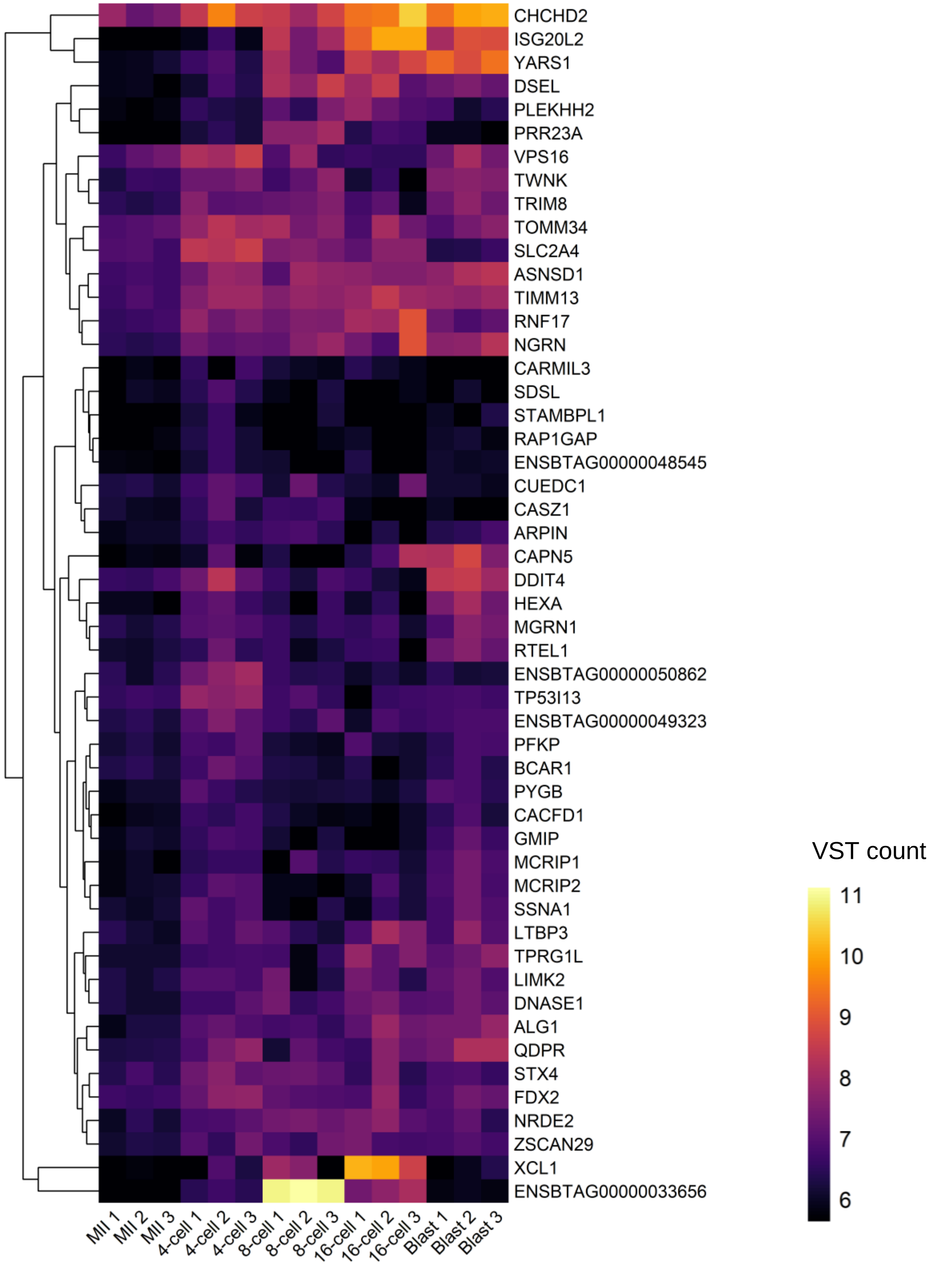

Supplementary Figure 6. Minor EGA products. VST normalized expression of genes that were differentially upregulated in 4-cell embryos relative to MII oocytes (n=62 genes; adjusted p-value < 0.05; log fold change in expression > 2). P-values derived by the Wald test and adjusted for multiple testing by the Benjamini-Hochberg method. RNA-seq data from Graf et al (2014) [1]. Source Data are provided as a Source Data file.

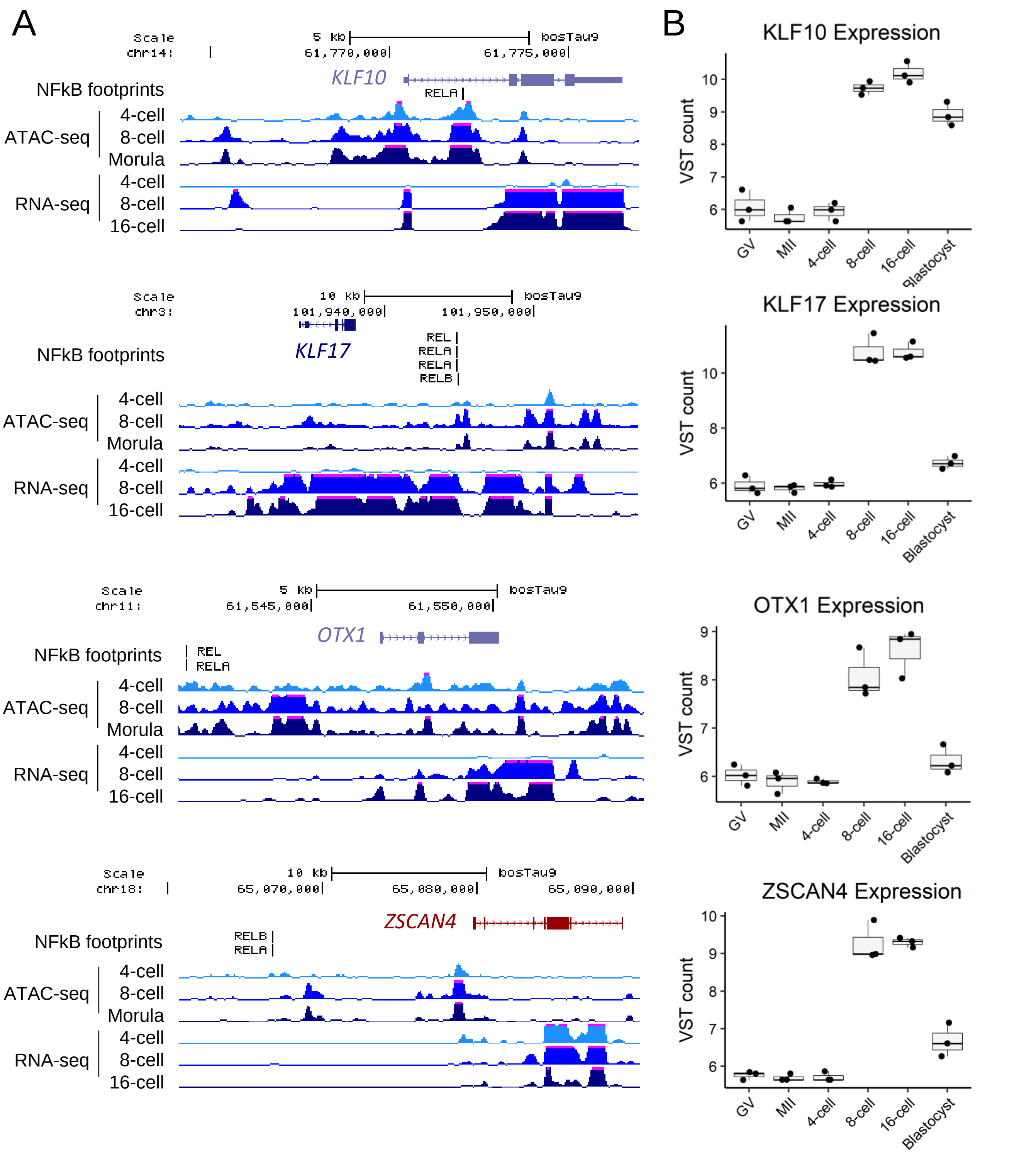

Supplementary Figure 7. Putative target genes of NFkB activation in 4-cell embryos. A) Accessibility and expression profiles of potential EGA regulators marked by NFkB factor footprints at the 4-cell stage. B) Normalized expression of the same genes in bovine oocytes and preimplantation embryos (n=3 biologically independent samples). Boxplots indicate the median and interquartile range (IQR), and whiskers span 1.5 times the IQR. RNA-seq data from Graf et al (2014) [1]. Source data are provided as a Source Data file.

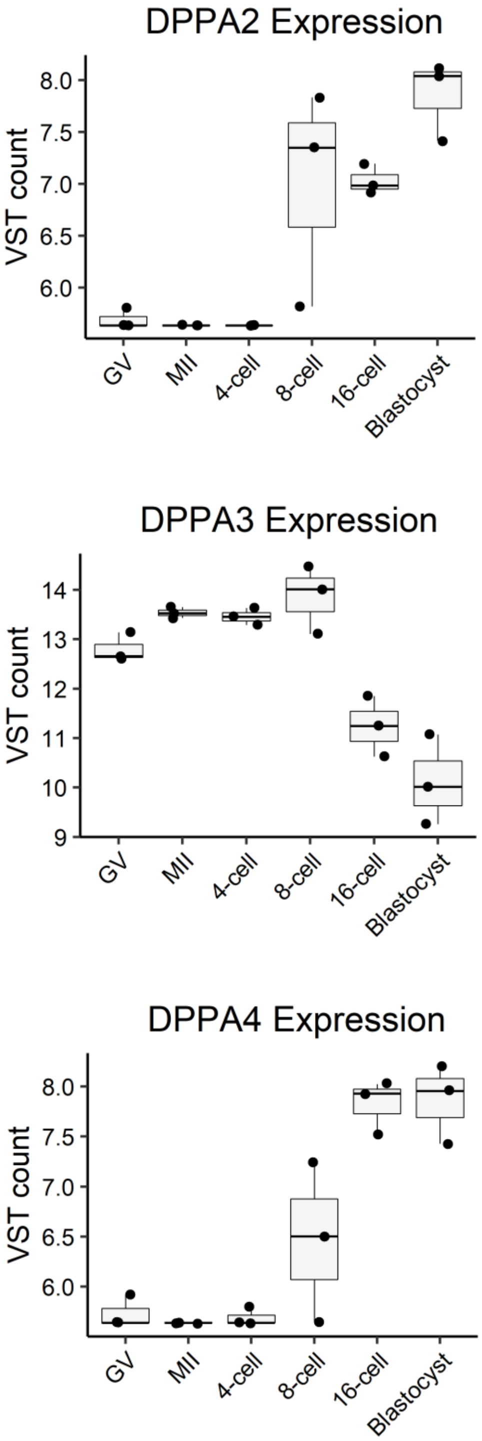

Supplementary Figure 8. VST expression profiles of DPPA genes during preimplantation development (n=3 biologically independent samples). Boxplots indicate the median and interquartile range (IQR), and whiskers span 1.5 times the IQR. RNA-seq data from Graf et al (2014) [1]. Source data are provided as a Source Data file.

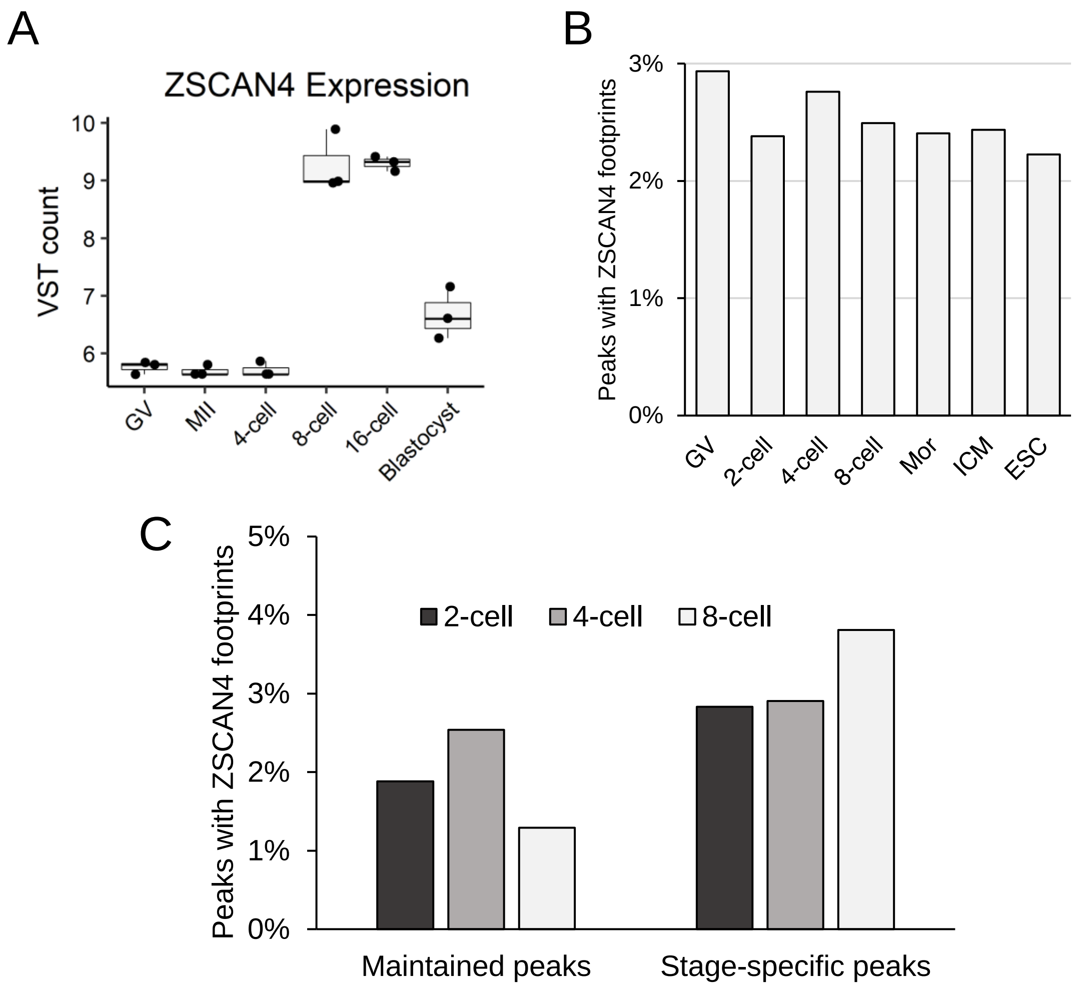

Supplementary Figure 9. A) VST expression profile of ZSCAN4 (n=3 biologically independent samples). Boxplots indicate the median and interquartile range (IQR), and whiskers span 1.5 times the IQR. B) Percent ATAC-seq peaks at each stage that contained ZSCAN4 footprints. C) ZSCAN4 footprints in stage-specific and maintained peaks at the 2-, 4-, and 8-cell stages. RNA-seq data from Graf et al (2014) [1]. Source data are provided as a Source Data file.

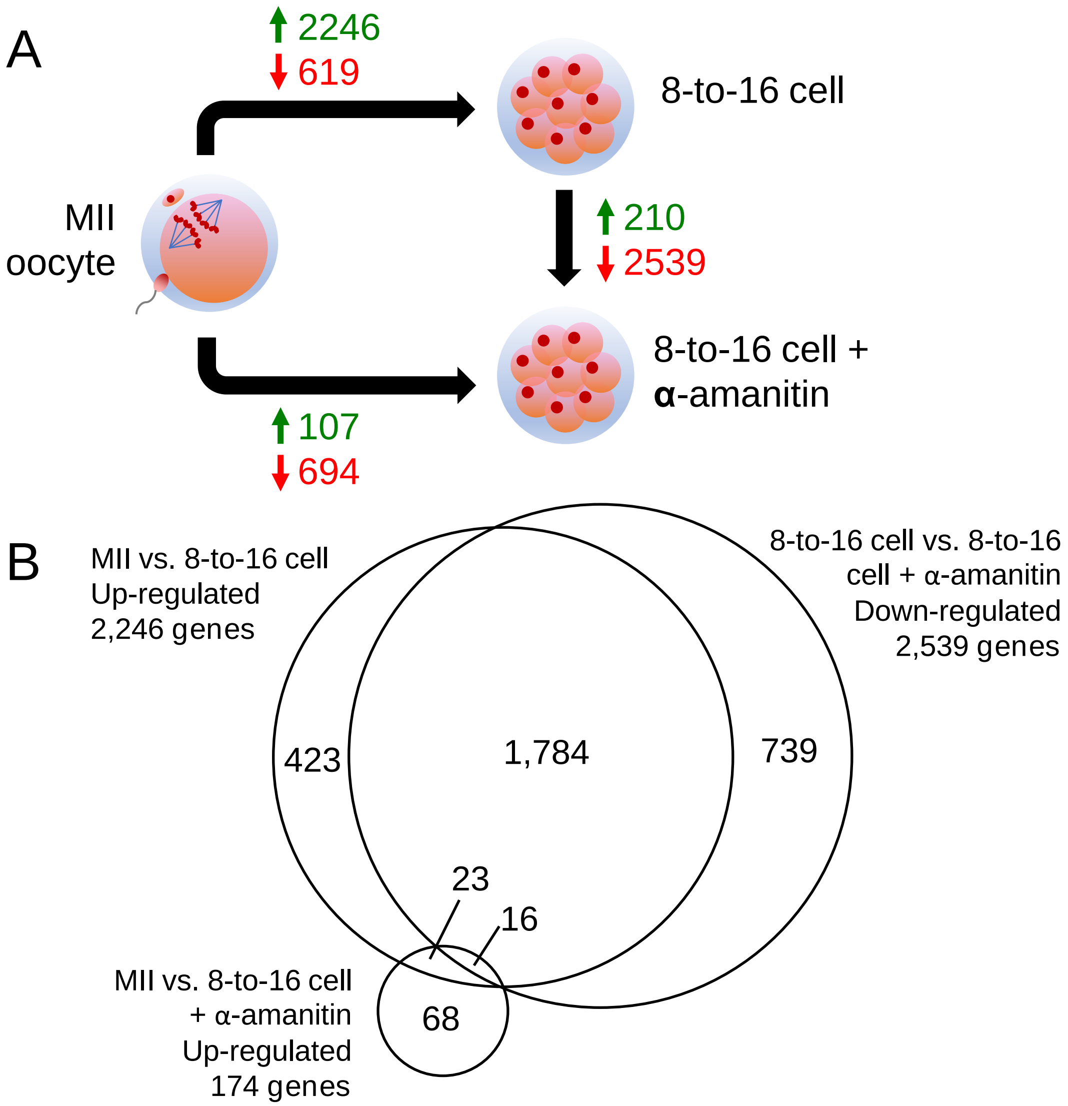

Supplementary Figure 10. Analysis of RNA-seq data from Bogliotti et al (2019) [2]. A) Differentially expressed genes (DEG) across developmental stages, which demonstrated an FDR < 0.05 and log fold change in expression > 2. P-values derived by the Wald test and adjusted for multiple testing by the Benjamini and Hochberg method. B) Venn diagram comparing DEG between different stages, allowing for the identification of 68 transcripts that were exclusively maternal in origin, and 1,784 transcripts that were exclusively embryonic in origin; i.e. EGA-specific genes. Source data are provided as a Source Data file.

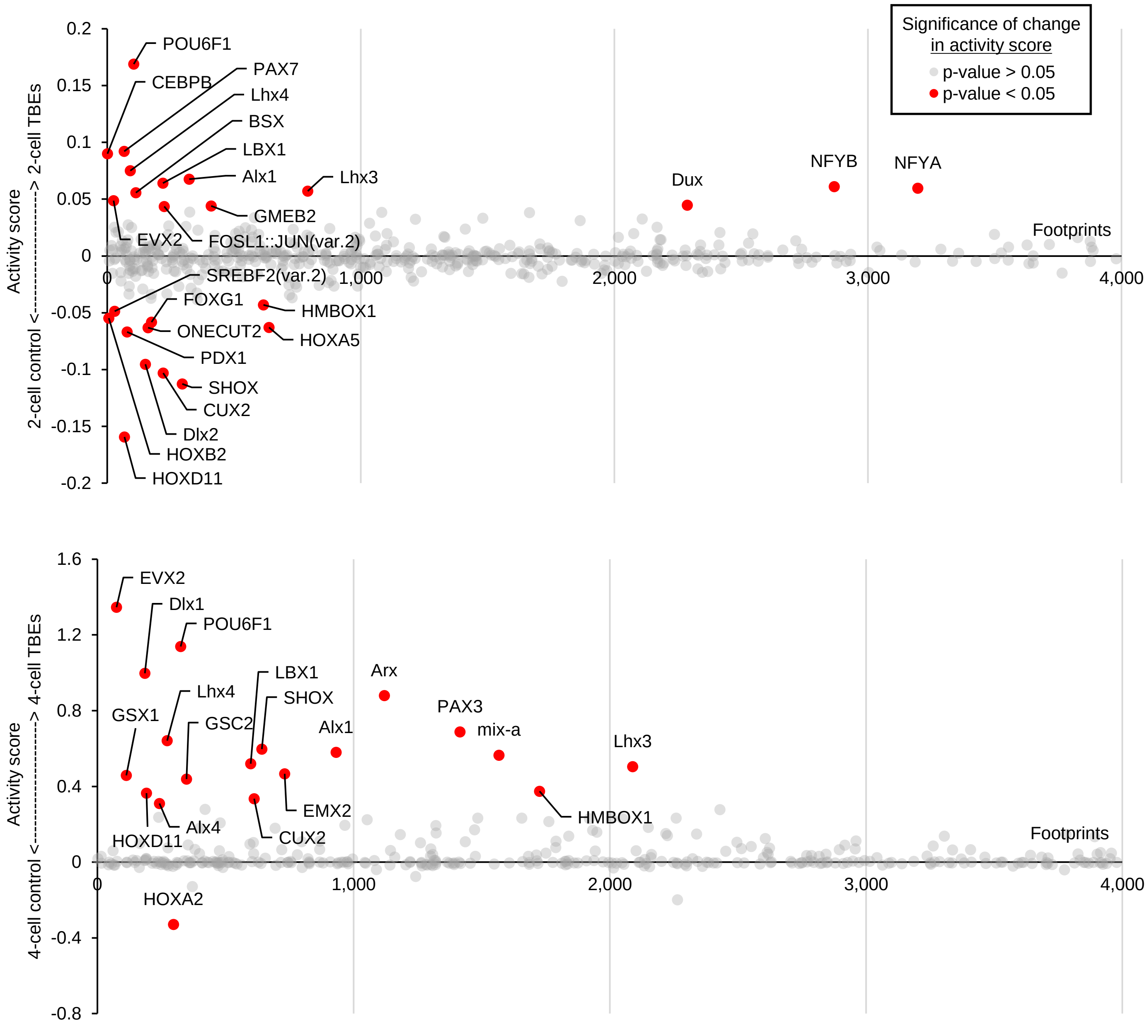

Supplementary Figure 11. Comparison of TF binding activity in control and TBE at the 2-cell and 4-cell stages. TFs colored in red demonstrated significantly increased activity (p-value <0.05; two-tailed t-test) in either TBE (positive activity score) or control embryos (negative activity score). Source data are provided as a Source Data file.

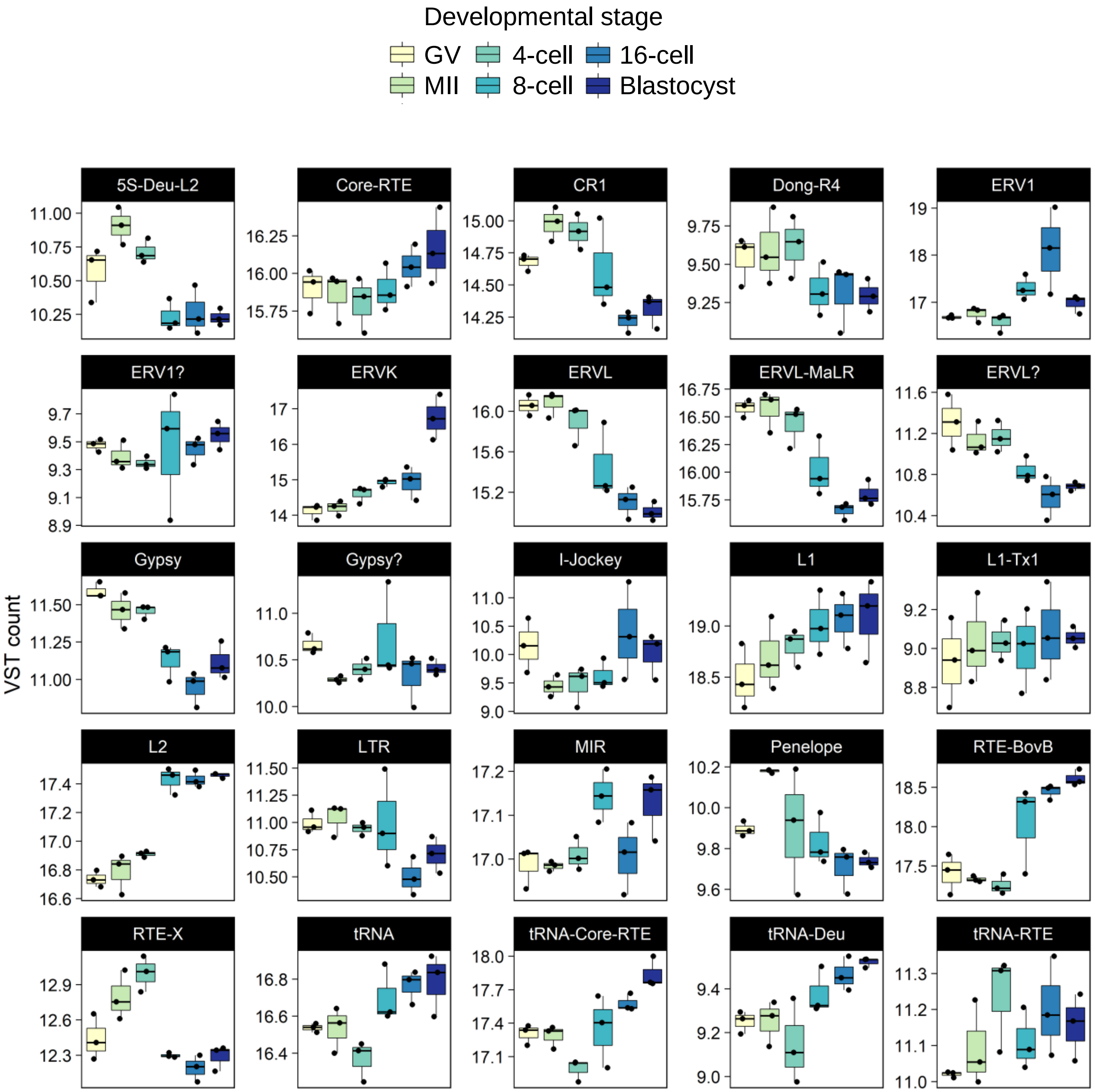

Supplementary Figure 12. Normalized expression profiles of all LTR, LINE, and SINE repeat families throughout bovine preimplantation development (n=3 biologically independent samples). Boxplots indicate the median and interquartile range (IQR), and whiskers span 1.5 times the IQR. Transcriptomic data generated for bovine oocytes and embryos by Graf et al (2014) [1]. Source data are provided as a Source Data file.

A

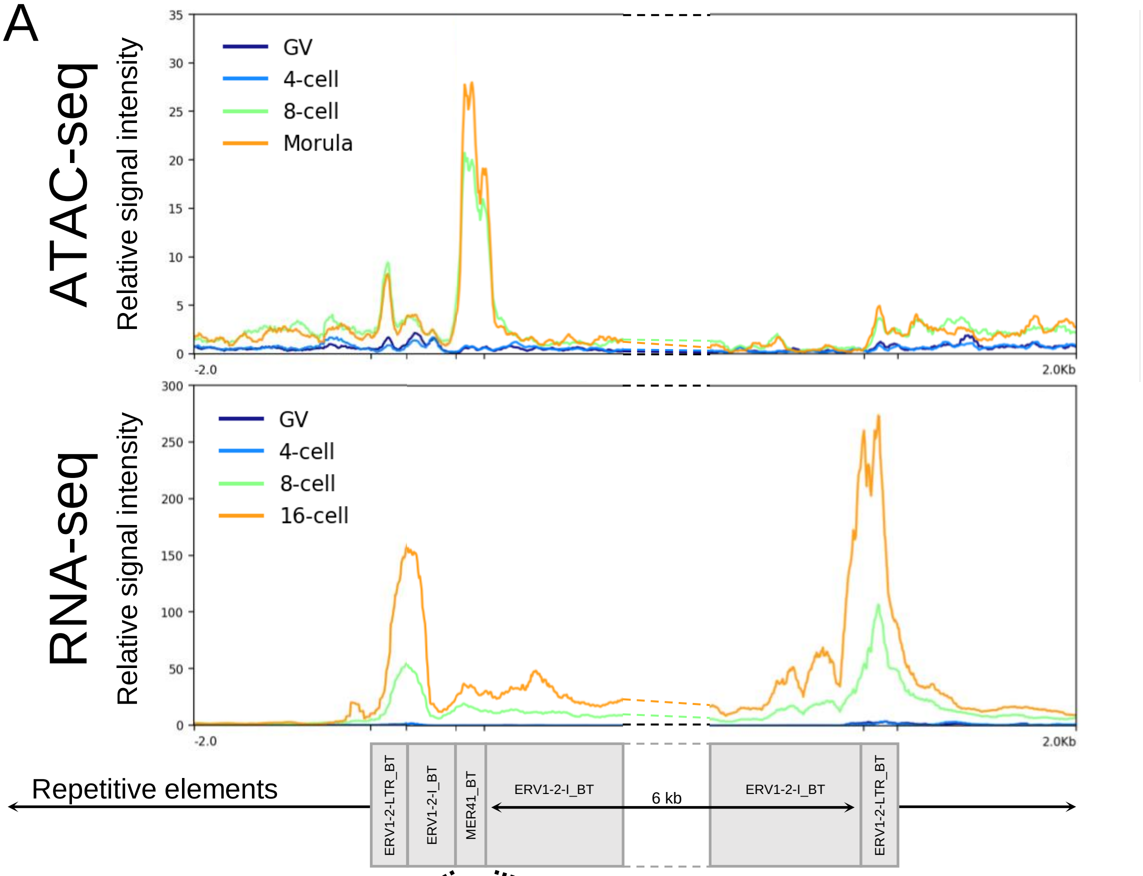

B

Motif	P-value	Open chromatin in MER41 with motif (%)
POU5F1-SOX2-TCF-NANOG	1e-20	29.4
POU5F1	1e-13	33.3
LHX2	1e-09	39.2
NFY	1e-07	33.3
KLF4	1e-07	23.5
OTX2	1e-07	31.4
TEAD	1e-06	31.4

Supplementary Figure 13. A) Average relative ATAC-seq (FPKM) and RNA-seq (RPKM) signal intensity at 57 ERV1-2-I_BT elements flanked by ERV1-2-LTR_BT with an internal MER41_BT element. B) Top known motifs enriched in open chromatin at MER41_BT elements that accompany ERV1-2-I_BT and ERV1-2-LTR_BT elements. Binomial test p-values reported (all FDR adjusted Q-values < 1e-4). RNA-seq data from Graf et al (2014) [1].

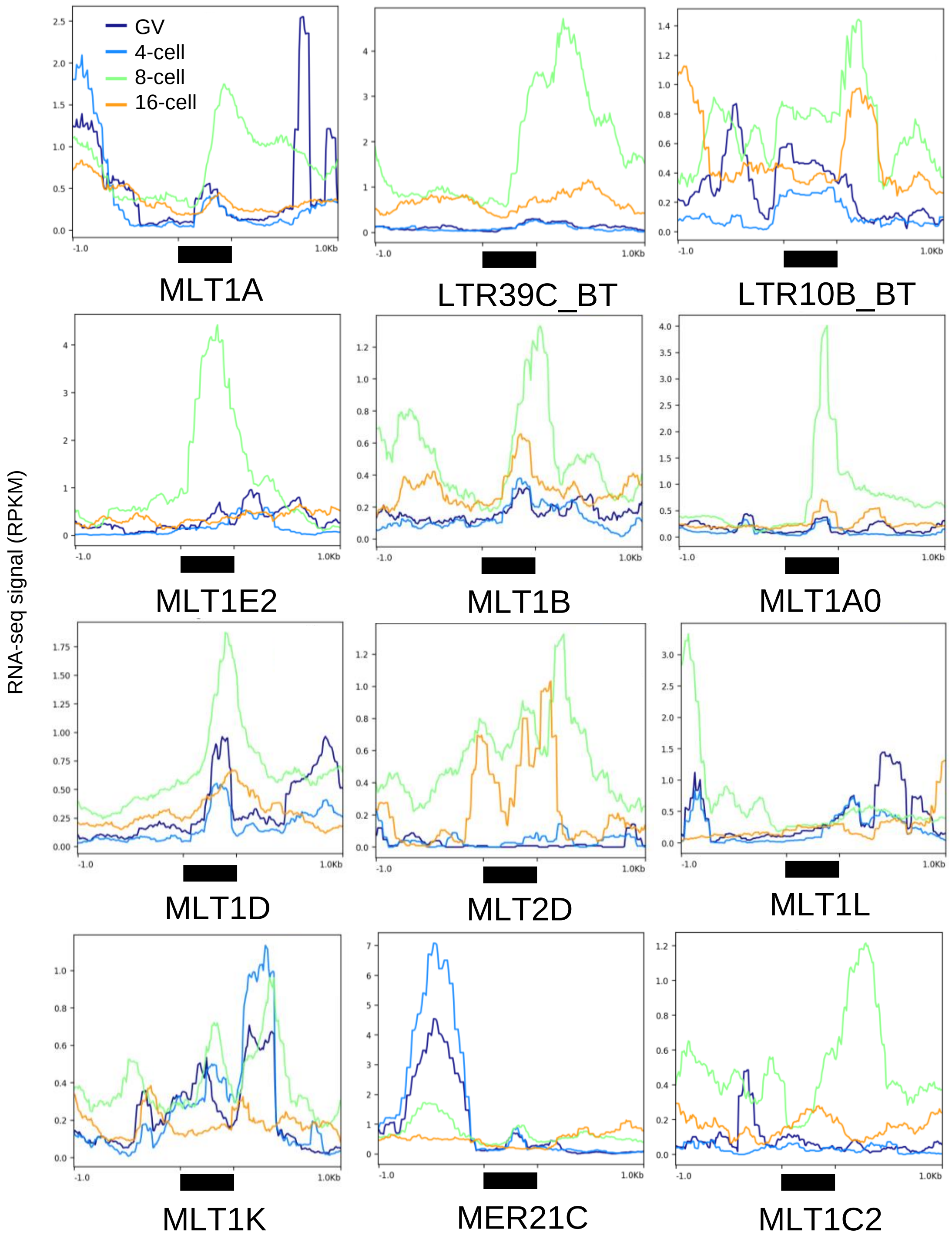

Supplementary Figure 14. Normalized RNA-seq signal (RPKM) at repeats overlapping distal 8-cell ATAC-seq peaks harboring DUXA motifs. RNA-seq from Graf et al (2014) [1].

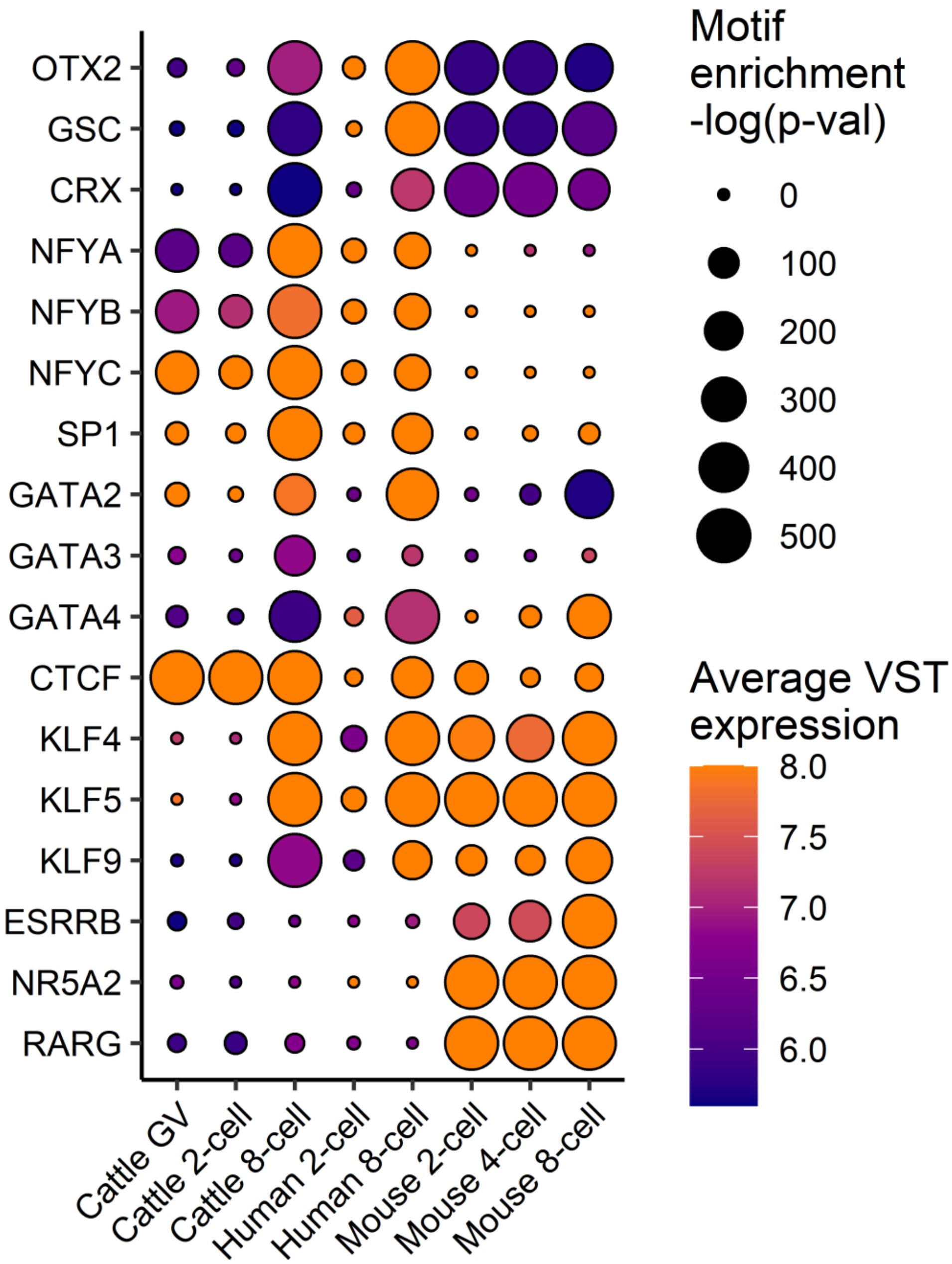

Supplementary Figure 15. Inference of key regulatory factors during preimplantation development in cattle, human, and mouse, based on enrichment for TF binding factors in open chromatin and expression of the corresponding TF. For motif enrichment, binomial test p-values reported. Bovine RNA-seq data from Graf et al (2014) [1]; human ATAC-seq and RNA-seq data from Wu et al (2018) [3]; mouse ATAC-seq and RNA-seq from Wu et al (2016) [4]. Source data are provided as a Source Data file.

Supplementary Table 1. Sequencing depth of individual ATAC-seq libraries. Total raw sequencing reads, percent alignment to the genome (excluding mitochondrial DNA), percent duplication among aligned reads, and informative (non-duplicate, non-mitochondrial uniquely mapping) reads.

Stage	Treatment	Rep.	Raw reads	Aligned reads (%)	Duplicate reads (%)	Informative reads	Total informative reads
GV oocyte	Control	1	237,846,070	56.92	90.71	7,520,394	30,191,548
		2	156,780,590	76.35	89.22	7,597,123	
		3	195,336,400	68.61	83.59	15,074,031	
2-cell	TBE	1	462,735,044	43.21	95.96	3,649,130	19,536,740
		2	67,607,200	59.97	77.60	5,792,638	
		3	113,594,712	67.41	80.08	10,094,972	
2-cell	Control	1	325,738,800	41.47	87.54	6,944,410	31,690,021
		2	284,762,902	47.72	93.78	4,044,907	
		3	107,281,710	72.97	61.13	20,700,704	
4-cell	TBE	1	758,041,924	43.26	94.17	10,208,325	19,198,081
		2	310,266,952	54.45	94.48	4,266,797	
		3	51,133,698	49.88	71.15	4,722,959	
4-cell	Control	1	744,499,540	50.11	92.99	14,825,045	36,653,439
		2	178,231,726	57.57	76.83	14,011,042	
		3	280,964,432	55.53	90.83	7,817,352	
8-cell	TBE	1	134,460,968	91.69	58.74	36,786,855	163,235,550
		2	538,922,446	85.69	65.42	116,170,135	
		3	100,844,568	50.38	71.33	10,278,560	
8-cell	Control	1	90,270,884	91.75	40.46	38,568,595	87,094,541
		2	180,127,326	56.53	69.97	23,632,106	
		3	216,383,872	52.26	71.43	24,893,840	
Morula	Control	1	127,179,516	73.94	81.43	13,744,880	64,586,045
		2	160,860,156	66.12	67.21	28,915,042	
		3	64,511,504	69.71	42.24	21,926,123	
ICM	Control	1	100,275,374	71.88	71.75	14,934,364	60,254,225
		2	182,505,258	67.93	83.57	13,259,741	
		3	152,238,016	79.56	65.02	32,060,120	
ESC	Control	1	45,150,240	45.30	68.62	5,139,322	36,726,659
		2	93,751,520	49.43	47.97	19,658,241	
		3	69,528,692	48.61	55.80	11,929,096	

Supplementary Table 2. Pearson correlation coefficients calculated for replicate ATAC-seq libraries from genome-wide coverage (FPKM in 500 bp windows).

		Pearson Correlation Coefficient			
Stage	Treatment	Rep. 1 – Rep. 2	Rep. 1 – Rep. 3	Rep. 2 – Rep. 3	Average
GV	Control	0.97	0.97	0.97	0.97
2-cell	Control	0.99	0.97	0.96	0.97
4-cell	Control	0.98	0.98	0.99	0.98
8-cell	Control	0.79	0.84	0.98	0.87
Morula	Control	0.93	0.95	0.96	0.95
ICM	Control	0.94	0.94	0.88	0.92
ESC	Control	0.92	0.97	0.97	0.95
2-cell	TBE	0.98	0.97	0.98	0.98
4-cell	TBE	1.00	0.99	0.99	0.99
8-cell	TBE	0.90	0.76	0.73	0.80

Supplementary Table 3. Top five de novo enriched motifs found in intergenic ATAC-seq peaks that were either unique to GV oocytes or gained between stages of development. Matching known motifs (log odds match score > 0.6) reported for each de novo motif. Binomial test p-values reported (all FDR adjusted Q-values < 1e-4).

	Rank	Matching known motifs	P-value	Loci with motif (%)	Background (%)
Peaks unique to GV oocytes	1	RFX4, RFX5, RFXDC2, RFX3, RFX1, RFX2	1e-191	23.83	15.93
	2	ZNF519, CRZ1, EGR3, EGR2, PITX1	1e-168	7.87	3.73
	3	No known matches	1e-157	6.50	2.91
	4	AP4, PTF1A, E2A, FIGLA, ASCL1, TCF21, TCF3, TCF12	1e-131	27.40	20.34
	5	ZBTB3, POU6F1	1e-114	3.59	1.39
Peaks gained from 2- to 4-cell	1	BORIS, CTCF, SP4, ZIC1, ZIC4	1e-830	5.01	0.80
	2	NFkB-p65, NFKB2, REL, NFkB-p50,p52, RELA, NFKB1	1e-132	10.49	7.04
	3	ZFP128, PLAGL1, ZNF354C, ZNF263, CRX, FOXH1	1e-126	0.22	0.00
	4	NKX3-1, ISL2, NKX3-2	1e-113	0.24	0.01
	5	ZFP691, ZNF263, MED1, SOX8, ZBTB3	1e-109	0.95	0.22
Peaks gained from 4- to 8-cell	1	DUX4, CRX, DMBX1, PITX1, PITX3, PAX7, GSC	1e-5781	35.17	9.81
	2	KLF5, KLF4, KLF1, GC-box, KLF14, KLF7, SP3	1e-1687	14.52	4.81
	3	NFYB, NFY, CCAAT-box, NFYA, DUX	1e-1543	11.57	3.44
	4	EWS-ERG, HOXC9, POU6F2	1e-1253	2.43	0.12
	5	RORgt, RORA	1e-999	1.90	0.09
Peaks gained from 8-cell to morula	1	KLF4, KLF5, KLF1, KLF7, GC-box, SP3, KLF14	1e-1375	20.21	9.84
	2	GATA4, GATA3, GATA5, GATA2, GATA1, GATA6	1e-1194	30.59	18.60
	3	CRX, OTX2, PITX3, PITX1, GSC, DMBX1, PITX2, OBOX6	1e-878	15.11	7.72
	4	AP-2gamma, AP-2alpha, TFAP2E, TFAP2C, TFAP2A, TFAP2B	1e-864	15.85	8.32
	5	BORIS, CTCF, SP4, ZIC1, ZIC4	1e-736	4.03	1.03
Peaks gained from morula to ICM	1	JUN-AP1, ATF3, AP1, JUNB, FOSL2, FOSL1, BATF, FRA1, JUND	1e-773	18.76	4.30
	2	GATA4, GATA5, GATA2, GATA1	1e-370	22.10	9.59
	3	EKLF, KLF1, KLF5, KLF4, KLF10, KLF9, SP3	1e-101	15.25	9.21
	4	No known matches	1e-98	0.51	0.01
	5	NKX2.2, GFX, ZBTB22, NKX2-1	1e-89	0.61	0.02
Peaks gained from ICM to ESC	1	SOX3, SOX10, SOX6, SOX15, SOX2, SOX14	1e-1053	24.16	13.74
	2	BORIS, CTCF, ZIC4, ZIC1	1e-895	11.62	5.10
	3	TEAD3, TEAD1, TEAD4, TEAD, TEAD2	1e-610	7.66	3.26
	4	ZIC1, ZIC3, ZIC2, ZIC, TCF21	1e-356	13.60	8.71
	5	TGIF1, MEIS1, MEIS2, TGIF1, MEIS3	1e-338	48.99	41.18

Supplementary Table 4. Functional enrichment of genes with promoters marked by open chromatin containing specific TF footprints. Gene ontology listed in order of significance.

Stage	TF family	Peaks with footprints	Marked promoters	Enriched gene ontology terms (FDR < 0.001)
GV	CTCF	7819	1747	None
	NFYs	5,242	2,043	protein binding, cell division, mitotic nuclear division, positive regulation of transcription (DNA-templated), negative regulation of nucleic acid-templated trasncription
	RFXs	5740	1213	protein binding, cilium assembly
4-cell	CTCF	6,754	1,434	None
	KLFs	23,920	7,494	protein binding, ATP binding, poly(A) RNA binding, protein serine/threonine kinase activity, cadherin binding involved in cell-cell adhesion, protein transport, cell-cell adhesion, protein phosphorylation, identical protein binding, protein complex binding, transcription coactivator activity, chromatin binding, intracellular protein transport
	NFKBs	7,392	1,541	Negative regulation of cell differentiation, negative regulation of transcription (DNA-templated), protein binding, poly(A) RNA binding
	NFYs	3,620	1,667	protein binding, negative regualtion of cell differentiation, cell division, mitotic nuclear division, ATP binding, G2/M transition of mitotic cell cycle
	SPs	23,405	8,135	protein binding, poly(A) RNA binding, ATP binding, protein serine/threonine kinase activity, protein phosphorylation, cell division, protein transport, negative regulation of transcription (DNA-templated), chromatin binding, cadherin binding involved in cell-cell adhesion, ubiquitin-protein transferase activity, identical protein binding, mitotic nuclear division, protein kinase activity, covalent chromatin modification, cell-cell adhesion, cell cycle, positive regulation of transcription (DNA-templated), ubiquitin protein ligase binding, intracellular protein transport, ligase activity, protein ubiquitination
8-cell	CTCF	8,123	1,447	protein binding
	DUXs	20,402	2,029	protein binding, negative regulation of cell differentiation, negative regulation of transcription (DNA-templated), poly(A) RNA-binding, cell division, mRNA splicing via spliceosome, ATP binding
	GSCs	9,242	744	negative regulation of cell differentiation
	KLFs	31,229	7,582	protein binding, poly(A) RNA binding, ATP binding, cell division, protein sertine/threonine kinase activity, intracellular protein transport, cadherin involved in cell-cell adhesion, mitotic nuclear division, cell-cell adhesion, protein phosphorylation, protein transport
	NFYs	10,402	2,225	protein binding, cell division, negative regulation of cell differentiation, nucleosome assembly, negative regulation of transcription (DNA-templated), ATP binding, unfolded protein binding
	OTXs	9,930	823	negative regualtion of cell differentiation
	PITXs	13,038	883	negative regulation of cell differentiation
	SPs	28,080	8,062	protein binding, poly(A) RNA binding, ATP binding, cell division, protein serine/ threonine kinase activity, cadherin binding involved in cell-cell adhesion, mitotic nuclear division, chromatin binding, transcription coactivator activity, kinase activity, ligase activity, DNA repair, cell-cell adhesion, regulation of cell cycle, cell cycle, protein kinase activity, negative regulation of transcription (DNA-templated), intracellular protein transport, protein phosphorylation, magnesium ion binding, SH3 domain binding, G1/S transition of mitotic cell cycle, transcription initiation from RNA polymerase II promoter, protein transport, regulation of signal transduction by p53 class mediator, protein dephosphorylation, protein complex binding
	ZSCAN4	3,148	544	Negative regulation of cell differentiation, positive regulation of cell proliferation, negative regulation of apoptotic process
Morula	CTCF	12,946	1,619	None
	GATAs	19,238	1,031	negative regulation of cell differentiation
	KLFs	58,804	9,119	protein binding, ATP binding, poly(A) RNA binding, cadherin binding involved in cell-cell adhesion, identical protein binding, cell-cell adhesion, protein serine/threonine kinase activity
	SPs	52,905	9,658	protein binding, poly(A) RNA binding, ATP binding, negative regulation of transcription (DNA-templated), protein serine/threonine kinase activity, cadherin binding involved in cell-cell adhesion, covalent chromatin modification, idnentical protein binding, kinase activity, cell-cell adhesion, positive regulation of transcription (DNA-templated), G1/S transition of mitotic cell cycle, ligase activity, vesicle-mediated transport, transcription coactivator activity, protein transport,, chromatin binding, protein phosphorylation, ubiquitin protein ligase binding, protein kinase binding, Wnt signaling pathway
ICM	CTCF	9,099	1,832	None
	GATAs	12,136	966	negative regulation of cell differentiation, negative regulation of apoptotic process, negative regulation of transcription (DNA-templated)
	JUNs	20,609	2,860	protein binding, negative regulation of cell differentiation, cell-cell adhesion
	KLFs	36,422	10,510	protein binding, poly(A) RNA binding, ATP binding, cadherin binding involved in cell-cell adhesion, protein serine/threonine kinase activity, cell-cell adhesion, negative regulation of transcription (DNA-templated), magnesium ion binding, identical protein binding, enzyme binding, protein phosphorylation, positive regulation of transcription (DNA-templated), negative regulation of transcription from RNA polyermase II promoter, positive regulation of transcription from RNA polymerase II promoter, protein transport, small GTPase mediated signal transduction, protein kinase binding
	SPs	34,768	11,363	protein binding, poly(A) RNA binding, protein serine/threonine kinase activity, ATP binding, cadherin binding involved in cell-cell adhesion, postiive regulation of transcription (DNA-templated), identical protein binding, positive regulation from RNA polymerase II promoter, protein phosphorylation, cell-cell adhesion, transcription coactivator activity, protein kinase binding, negative regulation of transcription from RNA polymerase II promoter, covalent chromatin modification, protein phosphorylation, negative regulation of transcription from RNA polymerase II promoter, covalent chromatin modification, protein autophosphorylation, negative regulation of transcription (DNA-templated), protein transport, intracellular signal transduction, G1/S transition of mitotic cell cycle, magnesium ion binding, protein kinase activity, small GTPase mediated signal transduction, transcription factor binding

Supplementary Table 5. Top enriched known motifs in ATAC-seq peaks that appeared at the 2-cell, 4-cell, or 8-cell stages, and which persisted up the morula stage. Binomial test p-values reported (all FDR adjusted Q-values < 1e-4).

	Motif	P-value	Peaks with motif (%)
Maintained 2-cell ATAC-seq peaks	NFY	1e-306	31.47
	CTCF	1e-158	13.54
	SP1	1e-152	41.11
	GFX	1e-126	4.15
	ETS	1e-109	21.62
	ELK1	1e-98	34.47
	ZBTB33	1e-98	8.28
Maintained 4-cell ATAC-seq peaks	CTCF	1e-2586	27.26
	BORIS	1e-1571	34.77
	SP1	1e-229	29.07
	NFY	1e-215	23.77
	ETS	1e-167	16.72
	KLF9	1e-156	27.69
	NRF1	1e-147	12.95
Maintained 8-cell ATAC-seq peaks	OTX2	1e-4190	45.31
	GSC	1e-3882	55.26
	CTCF	1e-2287	10.24
	KLF5	1e-1782	37.45
	CRX	1e-1758	71.94
	PHOX2A	1e-1734	24.04
	KLF4	1e-1595	15.48

Supplementary Table 6. Retrotransposon enrichment in 8-cell intergenic ATAC-seq peaks harboring DUXA motifs (n=16,804 loci). Retrotransposons that appeared in at least 100 ATAC-seq peaks are reported.

Repeat	Distal 8-cell peaks with DUXA motif	Background	Log ratio random to observed
MLT1A	507	32	2.763
LTR39C_BT	125	14	2.189
LTR10B_BT	239	28	2.144
MLT1E2	101	16	1.843
MLT1B	404	67	1.797
MLT1A0	443	74	1.790
MLT1D	512	87	1.772
MLT2D	108	19	1.738
MLT1L	185	34	1.694
MLT1K	311	58	1.679
MER21C	150	45	1.204
MLT1C2	197	65	1.109

Supplementary Table 7. Sequences of primers used for PCR amplification of ATAC-seq libraries. Primer names beginning with “2” contain indexes used for demultiplexing.

Primer	*Sequence (5’* to *3’)*
1	AATGATACGGCGACCACCGAGATCTACACTCGTCGGCAGCGTCAGATGTG
2A	CAAGCAGAAGACGGCATACGAGATCTAGTACGGTCTCGTGGGCTCGGAGATGT
2B	CAAGCAGAAGACGGCATACGAGATTTCTGCCTGTCTCGTGGGCTCGGAGATGT
2C	CAAGCAGAAGACGGCATACGAGATGCTCAGGAGTCTCGTGGGCTCGGAGATGT
2D	CAAGCAGAAGACGGCATACGAGATAGGAGTCCGTCTCGTGGGCTCGGAGATGT
2E	CAAGCAGAAGACGGCATACGAGATCATGCCTAGTCTCGTGGGCTCGGAGATGT
2F	CAAGCAGAAGACGGCATACGAGATGTAGAGAGGTCTCGTGGGCTCGGAGATGT
2G	CAAGCAGAAGACGGCATACGAGATCCTCTCTGGTCTCGTGGGCTCGGAGATGT
2H	CAAGCAGAAGACGGCATACGAGATAGCGTAGCGTCTCGTGGGCTCGGAGATGT
2I	CAAGCAGAAGACGGCATACGAGATCAGCCTCGGTCTCGTGGGCTCGGAGATGT
2J	CAAGCAGAAGACGGCATACGAGATTGCCTCTTGTCTCGTGGGCTCGGAGATGT
2K	CAAGCAGAAGACGGCATACGAGATTCCTCTACGTCTCGTGGGCTCGGAGATGT
2L	CAAGCAGAAGACGGCATACGAGATATCACGACGTCTCGTGGGCTCGGAGATGT
2M	CAAGCAGAAGACGGCATACGAGATACAGTGGTGTCTCGTGGGCTCGGAGATGT
2N	CAAGCAGAAGACGGCATACGAGATCAGATCCAGTCTCGTGGGCTCGGAGATGT
2O	CAAGCAGAAGACGGCATACGAGATACAAACGGGTCTCGTGGGCTCGGAGATGT
2P	CAAGCAGAAGACGGCATACGAGATACCCAGCAGTCTCGTGGGCTCGGAGATGT
2Q	CAAGCAGAAGACGGCATACGAGATAACCCCTCGTCTCGTGGGCTCGGAGATGT
2R	CAAGCAGAAGACGGCATACGAGATCCCAACCTGTCTCGTGGGCTCGGAGATGT
2S	CAAGCAGAAGACGGCATACGAGATCACCACACGTCTCGTGGGCTCGGAGATGT
2T	CAAGCAGAAGACGGCATACGAGATGAAACCCAGTCTCGTGGGCTCGGAGATGT
2U	CAAGCAGAAGACGGCATACGAGATTGTGACCAGTCTCGTGGGCTCGGAGATGT
2V	CAAGCAGAAGACGGCATACGAGATAGGGTCAAGTCTCGTGGGCTCGGAGATGT
2W	CAAGCAGAAGACGGCATACGAGATAGGAGTGGGTCTCGTGGGCTCGGAGATGT
2X	CAAGCAGAAGACGGCATACGAGATTCGCCTTAGTCTCGTGGGCTCGGAGATGT

Supplementary References

1. Graf, A. et al. Fine mapping of genome activation in bovine embryos by RNA sequencing. Proc. Natl. Acad. Sci. 111, 4139 LP – 4144 (2014).
2. Bogliotti, Y. S. et al. Transcript profiling of bovine embryos implicates specific transcription factors in the maternal-to-embryo transition. Biol. Reprod. (2019) doi:10.1093/biolre/ioz209.
3. Wu, J. et al. Chromatin analysis in human early development reveals epigenetic transition during ZGA. (2018) doi:10.1038/s41586-018-0080-8.
4. Wu, J. et al. The landscape of accessible chromatin in mammalian preimplantation embryos. Nature 534, 652–657 (2016).
