# Supplementary material for: Chromatin remodeling in bovine embryos indicates species-specific regulation of genome activation
Source: Nat Commun. 2020 Sep 17;11:4654. doi: 10.1038/s41467-020-18508-3 (PMC7498599; doi:10.1038/s41467-020-18508-3)
Supplement: Supplementary file 3 — Description of Additional Supplementary Files [file 41467_2020_18508_MOESM3_ESM.pdf]

Description of Additional Supplementary Files

File Name: Supplementary Data 1
Description: Genomic coordinates of TF footprints in bovine GV oocytes, with name of best matching JASPAR motif.

File Name: Supplementary Data 2
Description: Genomic coordinates of TF footprints in bovine 2-cell embryos, with name of best matching JASPAR motif.

File Name: Supplementary Data 3
Description: Genomic coordinates of TF footprints in bovine 4-cell embryos, with name of best matching JASPAR motif.

File Name: Supplementary Data 4
Description: Genomic coordinates of TF footprints in bovine 8-cell embryos, with name of best matching JASPAR motif.

File Name: Supplementary Data 5
Description: Genomic coordinates of TF footprints in bovine morula, with name of best matching JASPAR motif.

File Name: Supplementary Data 6
Description: Genomic coordinates of TF footprints in bovine ICM, with name of best matching JASPAR motif.

File Name: Supplementary Data 7
Description: Genomic coordinates of TF footprints in bovine ESC, with name of best matching JASPAR motif.

File Name: Supplementary Data 8
Description: Lists of genes that contained a footprint in their promoter for a given TF at a given developmental stage. For each gene, the Ensembl identifier and external gene name are provided. When available, the Ensembl identifier and external gene name of the human homolog is also included.
