# Supplementary material for: Chromatin remodeling in bovine embryos indicates species-specific regulation of genome activation
Source: Nat Commun. 2020 Sep 17;11:4654. doi: 10.1038/s41467-020-18508-3 (PMC7498599; doi:10.1038/s41467-020-18508-3)
Supplement: Supplementary file 12 — Reporting Summary [file 41467_2020_18508_MOESM12_ESM.pdf]

nature research

Corresponding author(s): *Double-blind peer review submissions: write DBPR and your manuscript number here instead of author names.*

Last updated by author(s): *YYYY-MM-DD*

Reporting Summary

Nature Research wishes to improve the reproducibility of the work that we publish. This form provides structure for consistency and transparency in reporting. For further information on Nature Research policies, see our Editorial Policies and the Editorial Policy Checklist.

Statistics

For all statistical analyses, confirm that the following items are present in the figure legend, table legend, main text, or Methods section.

n/a	Confirmed	
☐	☒	The exact sample size (*n*) for each experimental group/condition, given as a discrete number and unit of measurement
☐	☒	A statement on whether measurements were taken from distinct samples or whether the same sample was measured repeatedly
☐	☒	The statistical test(s) used AND whether they are one- or two-sided<br>*Only common tests should be described solely by name; describe more complex techniques in the Methods section.*
☒	☐	A description of all covariates tested
☐	☒	A description of any assumptions or corrections, such as tests of normality and adjustment for multiple comparisons
☐	☒	A full description of the statistical parameters including central tendency (e.g. means) or other basic estimates (e.g. regression coefficient) AND variation (e.g. standard deviation) or associated estimates of uncertainty (e.g. confidence intervals)
☒	☐	For null hypothesis testing, the test statistic (e.g. *F*, *t*, *r*) with confidence intervals, effect sizes, degrees of freedom and *P* value noted<br>*Give P values as exact values whenever suitable.*
☒	☐	For Bayesian analysis, information on the choice of priors and Markov chain Monte Carlo settings
☒	☐	For hierarchical and complex designs, identification of the appropriate level for tests and full reporting of outcomes
☐	☒	Estimates of effect sizes (e.g. Cohen's *d*, Pearson's *r*), indicating how they were calculated

Our web collection on statistics for biologists contains articles on many of the points above.

Software and code

Policy information about availability of computer code

Data collection	No software was used
Data analysis	Cutadapt (v0.4.0), BWA (v0.7.17), PicardTools (v2.8.1), SAMtools (v1.7), MACS2 (v2.1.1), Trimmomatic (v0.33), STAR (v2.7.2), GenomicAlignments (v1.18.1), DESeq2 (v1.22.2), TEtoolkit (v2.0.3), Deeptools (v3.2.0), BEDtools (v2.29.0), HOMER (v4.8), MEME (v5.0.4), DAVID (v6.8), RGT (v0.12.3)

For manuscripts utilizing custom algorithms or software that are central to the research but not yet described in published literature, software must be made available to editors and reviewers. We strongly encourage code deposition in a community repository (e.g. GitHub). See the Nature Research guidelines for submitting code & software for further information.

Data

Policy information about availability of data

All manuscripts must include a data availability statement. This statement should provide the following information, where applicable:
- Accession codes, unique identifiers, or web links for publicly available datasets
- A list of figures that have associated raw data
- A description of any restrictions on data availability

The following published data sets were used, and accessed through the NCBI GEO repository: for bovine oocytes and in vitro produced embryos, raw RNA-seq data were downloaded from accession number GSE5241567, and mouse and human preimplantation embryo ATAC-seq and RNA-seq data, raw sequencing files were downloaded from accession numbers GSE6639015 and GSE10157116, respectively. The ATAC-seq raw sequencing data produced in this study are available via the NCBI SRA repository under the SRA accession number PRJNA595394. Processed data files produced in this study are available via the GEO database under the accession number GSE143658.

Field-specific reporting

Please select the one below that is the best fit for your research. If you are not sure, read the appropriate sections before making your selection.

- [x] Life sciences
- [ ] Behavioural & social sciences
- [ ] Ecological, evolutionary & environmental sciences

For a reference copy of the document with all sections, see nature.com/documents/nr-reporting-summary-flat.pdf

Life sciences study design

All studies must disclose on these points even when the disclosure is negative.

Sample size	For each developmental stage/treatment group, cells from three separate oocyte collections were processed for ATAC-seq, generating three biological replicates. This permitted us to determine if any replicates deviated significantly from their groups.
Data exclusions	No data were excluded.
Replication	Normalized genome-wide ATAC-seq signal was compared between biological replicates through principal components analysis and correlated with the Spearman rank-order correlation coefficient. In general, replicates were consistent with one another, with the exception of libraries from 2-cell control embryos, which generally demonstrated lower enrichment than other developmental stages.
Randomization	From a given collection, oocytes were randomly divided into two groups: embryos to be cultured under control conditions, and embryos to be transcriptionally inhibited. Thus, for a given replicate, control and transcriptionally inhibited embryos were derived from the same oocyte collection, cultured concurrently, and harvested for ATAC-seq at the same developmental stage.
Blinding	It was not possible to blind during this study, as each replicate and developmental stage was collected separately and processed immediately. Data processing was not blinded; however, all sequencing data were processed through the same bioinformatics pipeline.

Reporting for specific materials, systems and methods

We require information from authors about some types of materials, experimental systems and methods used in many studies. Here, indicate whether each material, system or method listed is relevant to your study. If you are not sure if a list item applies to your research, read the appropriate section before selecting a response.

Materials & experimental systems

n/a	Involved in the study	
x		Antibodies
	x	Eukaryotic cell lines
x		Palaeontology and archaeology
x		Animals and other organisms
x		Human research participants
x		Clinical data
x		Dual use research of concern

Methods

n/a	Involved in the study	
	x	ChIP-seq
x		Flow cytometry
x		MRI-based neuroimaging

Eukaryotic cell lines

Policy information about cell lines

Cell line source(s)	Bovine ESC were derived from bovine ICM as described by Bogliotti et al (2018).
Authentication	Bovine source of cells was authenticated by karyotyping and genomic sequencing resulting from ATAC-seq. Cell pluripotency was determined by teratoma formation, and pluripotency factor immunostaining.
Mycoplasma contamination	All cell lines tested negative for mycoplasma contamination, based on PCR assay.
Commonly misidentified lines (See ICLAC register)	N/A

ChIP-seq

Data deposition

- [x] Confirm that both raw and final processed data have been deposited in a public database such as GEO.
- [x] Confirm that you have deposited or provided access to graph files (e.g. BED files) for the called peaks.

Data access links<br>*May remain private before publication.*	BED files are available as processed data files through GEO database.
Files in database submission	FASTA files (raw sequencing), BED files (peak calls), BIGWIG files (normalized genome-wide signal)
Genome browser session<br>(e.g. UCSC)	https://genome.ucsc.edu/s/mmhalstead/Bovine_embryo_ATACseq

Methodology

Replicates	Three biological replicates per developmental stage/treatment group. Comparing genome-wide normalized signal (FPKM) in biological replicates generally resulted in Pearson correlations > 0.9. Comparison of ATAC-seq peaks that were called from biological replicates resulted in Jaccard statistics of about 0.2 in earlier stages, and 0.3-0.4 in later stages.
Sequencing depth	Between 51 and 744 million raw reads were generated for each ATAC-seq library. After mapping and filtering, between 3.6 and 116 million non-mitochondrial monoclonal uniquely mapping reads were obtained for each ATAC-seq library. Overall, between 30 and 87 million non-mitochondrial monoclonal uniquely mapping reads were collectively obtained for each developmental stage, and at least 20 million non-mitochondrial monoclonal reads were collectively obtained for transcription blocked embryos (TBEs) at each stage.
Antibodies	No antibodies are required for the ATAC-seq protocol.
Peak calling parameters	Broad peaks were called with MACS2 (v2.1.1), using a q-value cutoff of 0.05, and settings --nomodel --shift -100 --extsize 200. For comparison between different stages or treatment groups, peaks were called from either 30 million (comparing developmental stages) or 20 million reads (comparing control and transcriptionally inhibited embryos).
Data quality	Calling peaks from 30 million reads per developmental stage resulted in between 14,916 (2-cell embryos) and 205,358 loci (morula-stage embryos) that were significantly enriched (q<0.05). Calling peaks from 20 million reads per developmental stage of transcriptionally blocked embryos resulted in between 46,650 (2-cell embryos) and 30,339 loci (8-cell embryos) that were significantly enriched (q<0.05). Average fraction of reads in peaks (a measure of signal) for replicate ATAC-seq libraries varied between 8.6% in control 2-cell embryos and 54.46% in morula-stage embryos.
Software	Raw sequencing reads were trimmed with Trim_Galore, a wrapper around Cutadapt (v0.4.0), to remove residual Illumina adapter sequences and low quality (q<20) ends, keeping unpaired reads and reads 10 bp or longer after trimming. Trimmed reads were then aligned the ARS-UCD1.2 assembly using BWA aln (-q 15) and sampe (v0.7.17). PCR duplicates were removed with PicardTools (v2.8.1), and mitochondrial and low-quality alignments (q<15) were removed with SAMtools (v1.7). Alignments from biological replicates from each stage were merged and randomly subsampled to equivalent depth with SAMtools for detection of open chromatin. To determine which regions of the genome demonstrated significant enrichment of ATAC-seq signal, broad peaks were called with MACS2 (v2.1.1), using a q-value cutoff of 0.05, and settings --nomodel --shift -100 --extsize 200. Processed alignments were converted to bigwig format using bamCoverage from the DeepTools suite, which binned the genome into 50 bp windows and calculated normalized signal (reads per kilobase million; RPKM) in each window. The plotPCA function from DeepTools was then used to generate principal components plots, with options –transpose and –log2. The plotCorrelation function from DeepTools was used to calculate the Spearman correlation coefficient between replicate libraries, based on genome-wide normalized coverage.
